# Hippocampal place cell encoding of sloping terrain

**DOI:** 10.1002/hipo.22966

**Published:** 2018-11-23

**Authors:** Blake S. Porter, Robert Schmidt, David K. Bilkey

**Affiliations:** ^1^ Department of Psychology University of Otago Dunedin, 9016 New Zealand; ^2^ Brain Health Research Centre Division of Sciences, University of Otago Dunedin, 9016 New Zealand; ^3^ Department of Psychology the University of Sheffield Sheffield, S1 2LT United Kingdom

**Keywords:** phase precession, place cells, three‐dimensional (3D) space

## Abstract

Effective navigation relies on knowledge of one's environment. A challenge to effective navigation is accounting for the time and energy costs of routes. Irregular terrain in ecological environments poses a difficult navigational problem as organisms ought to avoid effortful slopes to minimize travel costs. Route planning and navigation have previously been shown to involve hippocampal place cells and their ability to encode and store information about an organism's environment. However, little is known about how place cells may encode the slope of space and associated energy costs as experiments are traditionally carried out in flat, horizontal environments. We set out to investigate how dorsal‐CA1 place cells in rats encode systematic changes to the slope of an environment by tilting a shuttle box from flat to 15 ° and 25 ° while minimizing external cue change. Overall, place cell encoding of tilted space was as robust as their encoding of flat ground as measured by traditional place cell metrics such as firing rates, spatial information, coherence, and field size. A large majority of place cells did, however, respond to slope by undergoing partial, complex remapping when the environment was shifted from one tilt angle to another. The propensity for place cells to remap did not, however, depend on the vertical distance the field shifted. Changes in slope also altered the temporal coding of information as measured by the rate of theta phase precession of place cell spikes, which decreased with increasing tilt angles. Together these observations indicate that place cells are sensitive to relatively small changes in terrain slope and that terrain slope may be an important source of information for organizing place cell ensembles. The terrain slope information encoded by place cells could be utilized by efferent regions to determine energetically advantageous routes to goal locations.

## 1 INTRODUCTION

Navigation that accounts for the energetically‐demanding aspects of terrain topology has the potential to save an organism a great deal of time and energy compared to that which only considers the distance to a goal. In practical terms this is instantiated in the empirically validated (Scarf, 2007), century old, Naismith's rule (Naismith, 1892) for planning hiking routes: Account for 1 hr for every three miles (4,828 m) on flat terrain and one additional hour for every 2,000 feet (610 meters) of ascent. Over and above the costs associated with the extra time, humans (Hoogkamer, Taboga, & Kram, 2014; Margaria, Cerretelli, Aghemo, & Sassi, 1963; Minetti, Moia, Roi, Susta, & Ferretti, 2002) and rodents (Armstrong, Laughlin, Rome, & Taylor, 1983; Brooks & White, 1978; Chavanelle et al., 2014) expend significantly more energy when travelling on inclined surfaces compared to travelling on flat ground. Many other species, including elephants (Wall, Douglas‐Hamilton, & Vollrath, 2006) and monkeys (Di Fiore & Suarez, 2007) appear to factor in these time and energy costs when navigating, as they avoid traversing over hills in their natural habits when alternatives are available. In particular, monkeys will travel along energetically advantageous “highways” year after year, suggesting that they possess a representation of the environment that includes the effort demands of routes (Di Fiore & Suarez, 2007). However, while topology‐related factors clearly influence navigation, it is not clear how the brain represents the potentially costly three‐dimensional (3D) nature of ecological environments (Jeffery, Jovalekic, Verriotis, & Hayman, 2013).

Place cells (O'Keefe & Dostrovsky, 1971; O'Keefe & Nadel, 1978) are hippocampal neurons that appear to have a role in representing the spatial environment. These cells are active at a specific location in an environment such that an ensemble of many place cells will encode an entire region as well as many features of that environment (for review; Eichenbaum, 2004). A diverse range of external sensory inputs have been shown to modulate and drive the selective firing of place cells such as environmental contexts (Muller & Kubie, 1987; Smith & Mizumori, 2006), landmarks (Gothard, Skaggs, Moore, & McNaughton, 1996; Knierim, Kudrimoti, & McNaughton, 1998), objects (Komorowski, Manns, & Eichenbaum, 2009; McKenzie et al., 2014), and odors (Jeffery & Anderson, 2003). The vestibular system also has a part to play as lesions of this region abolish the spatial selectivity of place cells (Russell, Horii, Smith, Darlington, & Bilkey, 2003; Stackman, Clark, & Taube, 2002) as well as impair spatial memory and navigation (Smith, 1997; Smith et al., 2005). These findings suggest that self‐motion (Wallace, Hines, Pellis, & Whishaw, 2002), gravitational, and head/body orientation (Stackman & Taube, 1997; Taube, 1998) information provided by the vestibular system are vital to a place cell's functionality and the neural representation of space.

As a result of their vestibular inputs, place cells may be especially attuned to gravitational and head/body orientation information which may allow them to encode the topology space. Previous studies have, for example, shown that place cell activity is sensitive to changes in slope. For example, when half of a rectangular track was tilted (Knierim & McNaughton, 2001), some place cells altered their activity by firing in a different location or shutting off all together with new place cells becoming active; a phenomenon known as “remapping.” In a separate study (Jeffery, Anand, & Anderson, 2006) it was shown that the rotation of a tilted open field caused the ensemble of place cells that represented the field to shift their fields in relation to the rotation, indicating that the cells were sensitive to the slope direction.

Despite these findings, it remains unclear how place cells, and ultimately the cognitive maps that might be used for navigation, encode terrain slope. Previous experiments investigating this question (Jeffery et al., 2006; Knierim & McNaughton, 2001) used steep slope angles which did not allow for the full investigation of the cell's sensitivity to terrain slope; are place cells responsive to small changes in slope angle or do they require a substantial slope to alter their firing patterns? Furthermore, the latter study (Knierim and McNaughton, 2001) is the only previous investigation where the slope of the environment was changed systematically. Unfortunately, in this previous experiment changes to the tilt of the apparatus were accompanied by changes to the rat's view of the external environment which may have confounded any effects observed.

More generally, debate continues as to whether or not land travelling mammals encode the vertical axis of space (Taube & Shinder, 2013). One proposal is that encoding of height within a cognitive map is minimal (Hayman, Verriotis, Jovalekic, Fenton, & Jeffery, 2011) and that multiple planar maps are used to represent each surface which are then pieced together to encode 3D space (Jeffery et al., 2013). Alternatively, it has been proposed that mammalian brains may be capable of encoding space in different ways depending on the environment and how an organism travels through it (Savelli & Knierim, 2011; Ulanovsky, 2011). For example, surface locomotion may result in the generation and use of anisotropic (vertical space is encoded differently than horizontal space) planar maps (Ulanovsky & Moss, 2007) while flying results in the use of isotropic (horizontal and vertical space are encoded in the same manner) volumetric maps (Finkelstein et al., 2015; Yartsev & Ulanovsky, 2013).

In the present experiment we set out to gain a better understanding of how rodent place cells respond to and represent tilted surfaces and in doing so to shed light on how cognitive maps encode three‐dimensional space. We recorded place cells from dorsal CA1 as rats ran back and forth on a cue‐devoid linear track which could either lie flat (0 °) or be tilted to 15 ° and 25 °. Our data show that place cells were sensitive to as little as 10 ° (15 ° to 25 °) changes in tilt and partial remapping was observed between all tilt conditions. Furthermore, the amount of remapping observed was positively correlated with how different the angle was between any two conditions. Nonetheless, a subgroup of place cells also remained stable across tilt conditions, continuing to represent a location on the track, irrespective of slope. Together, these data suggest that the firing of a subpopulation of place cells is modulated by the slope of an environment with individual place cells having different levels of sensitivity to slope angle. We also provide further evidence that the rat, a land‐travelling mammal, utilizes an anisotropic encoding scheme for representing 3D space.

## 2 MATERIALS AND METHODS

### 2.1 Subjects

Seven male Sprague Dawley rats were aged between 4 and 6 months old and weighed between 350‐500 grams were obtained from the University of Otago's Hercus‐Taieri Resource Unit. Upon arrival rats were housed in groups of three. Grouped rats were housed in plastic cages with wire metal lids (40 × 55 × 27 cm^3^). The animal housing room was maintained at a 12 hr light/dark cycle and kept between 20 and 22 °C. Rats were given 2 weeks from the time of arrival to acclimate to the new facility where they had *ad libitum* access to food (18% Protein Rodent Diet, Teklad Global) and water. After 2 weeks, rats were food deprived to no less than 85% of their free‐feeding weight to stimulate interest in the food reward (Coco Pops cereal, Kellogg Company) used for training and given in the experimental phase. Water continued to be available *ad libitum* throughout the study. All experimentation was done during the light phase.

### 2.2 Apparatus

The experiment was conducted in a wooden shuttle box measuring 120‐cm long by 24‐cm wide with 60‐cm tall walls. The entire apparatus was painted matte black and was devoid of any visual cues. The floor was a matte black rubber mat with a diamond pattern to provide the animals with grip while running. At each end of the shuttle box was a matte black plastic semi‐circular well where the food reward (Coco Pops) was dispensed. The Coco Pops were delivered through a PVC tube so that the experimenter could unobtrusively provide the rat with a food reward without interfering with cues inside the box.

**Figure 1:**
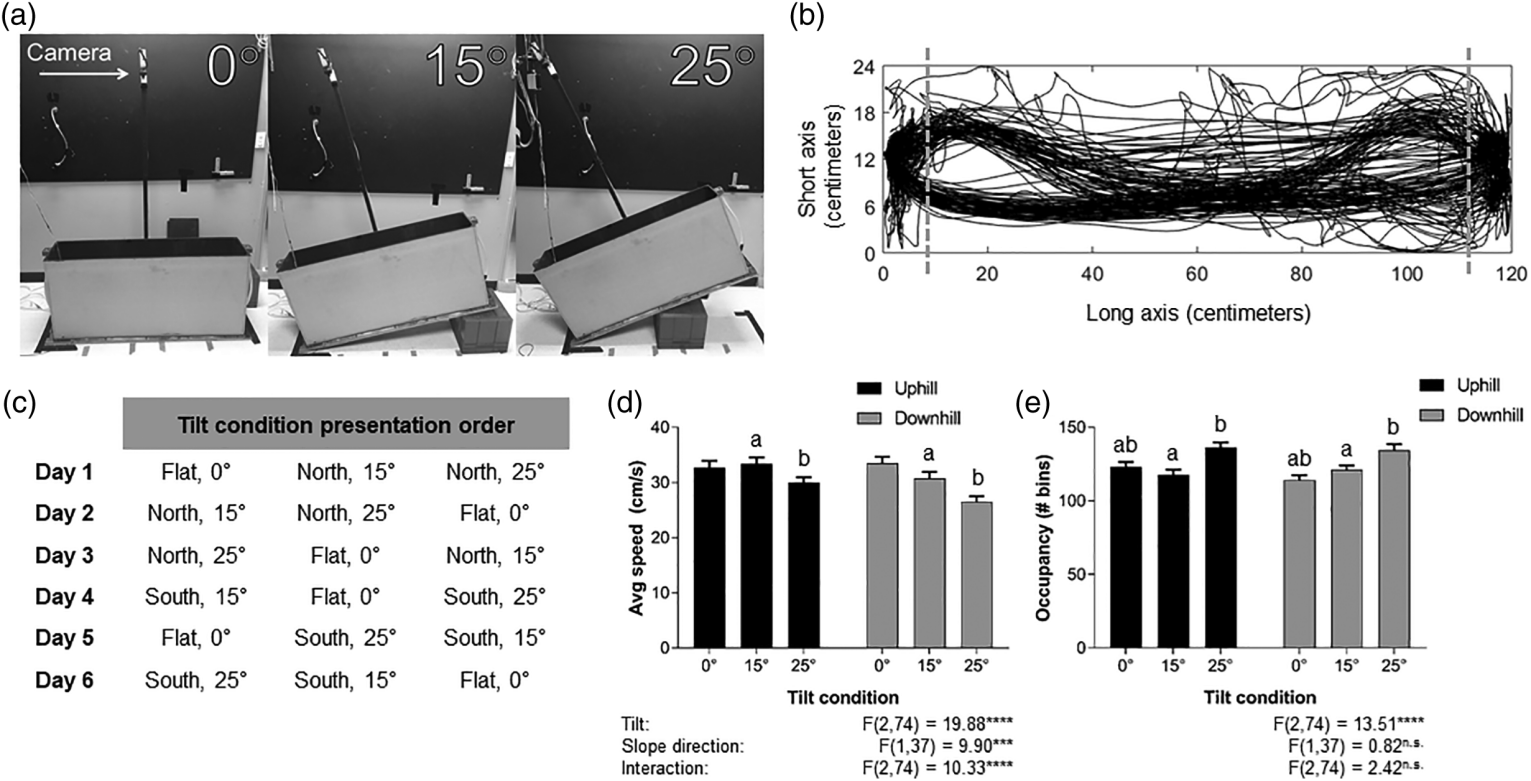
Experimental setup and behavioral results. (a) Pictures of the experimental apparatus at the three tilt conditions with the location of the camera marked. (b) Schematic of the apparatus, the boundaries (dashed line) of the running region of interest and two endzones. The solid black tracing is the tracking data from one recording session showing the rat's running pattern. (c) Experimental sequence. Rats were run for six consecutive days with tilt condition presentation order counterbalanced across days. (d) Average running speed of the rats across all tilt‐slope direction conditions. Rat's speed slowed with increasing tilt angle and was slowest for downhill runs. Bars sharing the same letter are significantly different from one another. (a) Tukey's, *p* = 0.004 between uphill 15 ° and downhill 15 °; (b) Tukey's, *p* < 0.001 between uphill 25 ° and downhill 25 °. (e) The amount of space rats utilized while shuttling. Rat's tended to take more irregular routes on tilted conditions. Bars sharing the same letter are significantly different from one another. (a) Tukey's, *p* < 0.0001 between 0 ° and 15 °; (b) Tukey's, *p* < 0.0001 between 0 ° and 25 °

The apparatus could be laid flat on the ground so that the floor of it was horizontal (0 °), and also tilted to two different inclines, 15 ° and 25 ° (Figure 1a). A camera was used to record the position of the rat based on infrared LEDs fixed to the data acquisition system's headstage. This camera was mounted to the apparatus at its midpoint so that its field of view of the maze remained constant when the apparatus was tilted. To minimize any extra‐maze cues, the only source of light in the room was a computer monitor 2.3 m away from the apparatus. The monitor's brightness was dimmed as low as possible. All other sources of light in the room were covered including the LEDs on equipment and the door jambs. Furthermore, the wall closest to the apparatus was painted matte black, as seen in Figure 1a. The two walls perpendicular to the apparatus, the only two possibly viewable by the rats when the apparatus was tilted, were both over a meter away and devoid of any cues. Because of the measures that were used to minimize extra‐maze cues, combined with the known poor visual acuity of albino rats (Prusky et al., 2002), it is extremely unlikely the rats could detect any visual changes associated with tilt.

### 2.3 Preoperative training

During the first 5 days of preoperative training, rats were familiarized to the recording room, experimenter, and apparatus. Rats were placed in the experimental apparatus and allowed to free forage for Coco Pops randomly scattered throughout. Once rats were readily foraging in the apparatus they were encouraged to shuttle between the two endzones by making Coco Pops only available at either end. When rats were readily shuttling while the apparatus was at 0 ° we began to tilt the apparatus. Initially, rats were allowed to shuttle for five minutes with the apparatus at 0 ° and given two Coco Pops upon arrival at the endzone. The apparatus was then tilted to 15 ° with the rat still in the apparatus. The rat then shuttled at 15 ° for five minutes for the same reward amount. Following this, the apparatus was tilted to 25 ° and the rat shuttled for a third 5‐min session with the same reward amount. Each day the side of the apparatus which was elevated was alternated. Some rats shuttled in the tilted conditions on the first day of exposure while others took up to 7 days to shuttle in all tilt conditions. Once a rat was shuttling for five minutes in each condition, a lap count measure of performance was utilized. A lap consisted of the rats running from one endzone to the opposite endzone and then back. Rats were trained each day until they were readily shuttling for 20 laps in each of the three tilt conditions. It took an average of 2 days for rats to reach the 20 lap criterion. All rats were then run on the 20 lap per condition sequence for at least 1 week to ensure consistent behavior (no stopping or turning around during a lap). At this point rats were ready to be implanted with microdrives.

### 2.4 Surgery

All experimental protocols were approved by the University of Otago Animal Ethics Committee and conducted in accordance with New Zealand animal welfare legislation. Anaesthesia was induced through 5% isoflurane (Merial New Zealand) in oxygen and maintained at 2–2.5% during surgery. Once induced, animals were given the analgesics Carprofen (a nonsteroidal anti‐inflammatory drug, 1 mg kg^−1^) and Temgesic (buprenorphine, 0.33 mg kg^−1^) as well as a prophylactic antibiotic, Amphoprim (trimethoprim and sulfamethazine, 0.2 mL,) before being placed into a stereotaxic frame with non‐puncture ear bars (David Kopf Instruments). The scalp was shaved and sterilized with Betadine (Povidone‐iodine) followed by a subcutaneous injection of Lopaine (lignocaine hydrochloride 20 mg mL^−1^; 0.1 mL diluted in 0.4 mL of sterile saline) as a local anesthetic in the scalp.

Six rats were implanted with 8‐channel Scribe microdrives (Bilkey & Muir, 1999) and one rat was implanted with a custom 64‐channel microdrive array. The electrodes of all drives were prepared as tetrodes (four electrodes tightly spun and heated together); two tetrodes for the 8‐channel drive and 15 for the 64‐channel drive. Electrodes consisted of 25 µm nichrome, heavy formvar insulated wire (Stablohm 675 HFV NATRL; California Fine Wire Company) for Scribe microdrives. For the 64‐channel microdrive array, the electrodes were made from 17.5 µm platinum 10% iridium, polymide insulated wire (California Fine Wire Company). All electrodes were gold (nichrome) or platinum (platinum 10% iridium) electroplated to reduce their impedances to between 200 and 250 kΩ (NanoZ; Neuralynx). Tetrodes were stereotaxically targeted at the dorsal aspect of the hippocampal CA1 subregion of the right hemisphere [anteroposterior, −3.7 mm; mediolateral, + 2.2 mm (Paxinos & Watson, 2007)]. Tetrodes were lowered ∼1.5 mm from the dura into the brain. Rats were also implanted with a single local field potential (LFP) electrode (200‐µm thick insulated nichrome wire; Johnson Matthey) into the ACC (anteroposterior, + 2.0 mm; mediolateral, + 0.4 mm). The ACC LFP data is not presented in this article. A skull screw over the cerebellum served as a ground connection. Post‐surgery rats received secondary doses of Temgesic, Carprofen, and Amphoprim. Rats were given 10 days to recover before behavioral testing resumed.

### 2.5 Postoperative training

Postoperative training was carried out to ensure rats could still perform the task adequately, adjust to their implant, and to optimize electrode placement. Rats' food was again reduced to maintain 85% of their free feeding weight. For each day of postoperative training rats were plugged into the data acquisition system's tethered headstage. On the first day of postoperative training, Coco Pops were randomly placed within the apparatus and the rat was given 15 min to forage freely to adjust to the weight of their implant. On subsequent days, rats shuttled for 20 laps on each tilt condition, 0 °, 15 °, and 25 °, counterbalancing for which end of the apparatus was elevated. Nearly all rats were able to carry this out on the first day. However, a few rats took between three to 5 days to acclimate to their implants and carry out all 60 laps. All rats had a minimum of seven sessions of postoperative training (20 laps per condition) prior to starting the experimental protocol.

### 2.6 Electrophysiological recordings

During postoperative training single unit and local field potential (LFP) data were closely monitored. Tetrodes were lowered towards dorsal CA1 (dCA1) over the course of 2 to 4 weeks until well isolated single units were identified. During this period rats were running the postoperative training outlined above. Neurophysiological and animal movement data were acquired with an Axona multichannel data acquisition system (DacqUSB; Axona, Ltd.) for both the 8‐ and 64‐ channel microdrives. Single unit data was bandpass filtered between 600 and 6,000 Hz and digitized at 48 kHz. Signals were amplified between 5,000 and 9,000 times. For each tetrode, one electrode with minimal spiking activity on a different tetrode served as a reference. Action potentials were detected by threshold crossing of ∼70 µV. LFP data was sampled at 4,800 Hz and bandpass filtered between 1 and 500 Hz.

### 2.7 Experimental protocol

Once dCA1 single units were being consistently obtained day to day the experimental sequence began. The experimental sequence consisted of six recording sessions, one per day for 6 days. Each day rats consecutively ran ∼20 laps in each of the three tilt conditions, 0 ° flat, 15 ° tilt, and 25 ° tilt. One lap consisted of the rat running from one end of the shuttle box to the other, consuming the reward at the endzone, and returning to the start endzone to consume its reward. After ∼20 laps under one condition, the rat remained in the apparatus and ran one more lap while the apparatus was tilted to the next condition. Tilt condition presentation order was counterbalanced across days such that no condition was experienced in the same order position (Figure 1c) and for the first 3 days the north endzone was elevated, while for the second 3 days the south endzone was elevated.

During the experimental sequence tetrodes were not manipulated. Three rats were run on the whole experimental sequence once while three were run on it twice with at least a 2‐day break in between data collection. In between the two 6‐day data collection sequences tetrodes were manipulated in order to obtain recordings from new single units. Tetrodes were lowered ∼40–80 µm per day until new units were obtained (visual inspection of waveforms online and offline) or until the tetrodes moved out of the dCA1 layer.

### 2.8 Analysis

For each recording, single units were manually isolated offline in Offline Sorter (Version 3; Plexon) primarily using peak‐to‐valley distance and principal components analysis of the waveforms. The single unit spiking data was then exported to Matlab along with the behavioral tracking data. All data analysis was carried out using Matlab with native and custom written scripts. All measurements are stated as means ± standard error of the mean.

### 2.9 Behavior analysis

The apparatus was broken up into two regions of interest (ROIs); running and endzones (Figure 1b). The endzones consisted of the two ends of the shuttle box where the reward was dispensed and consumed. The area in between the two endzone boundaries (103 cm long) was considered the running ROI where rats were actively shuttling between endzones. All analysis reported here was restricted to the running ROI. One trial counted as the rat running from the boundary of one endzone to the boundary of the opposite endzone (half a lap). Trials where the rat did not complete the end to end run were excluded. Failed shuttles were, however, quite rare, typically occurring on only one to two trials per condition. Trials where the rat took longer than 7 s to shuttle (had an average speed below 15 cm s^−1^) were also excluded to keep trial‐to‐trial speeds consistent. A trial's slope direction was determined to be uphill or downhill based on which endzone the rat departed from. Trials where the rat originated from the endzone on the ground and shuttled to the elevated endzone were considered uphill and vice versa for downhill. For the non‐tilt (0 °) condition, “uphill” and “downhill” trials correspond to the same running direction in relation to the tilted conditions occurring during that session.

### 2.10 Single unit analysis

For every single unit, the firing rate of each trial was determined by the duration of the trial and the number of spikes that cell fired during that trial. Condition (tilt by slope direction) firing rates were determined by dividing total trial durations for that condition by the number of spikes that occurred in that condition. All analyses were restricted to cells categorized as place cells. To be considered a place cell, single units had to have discharged at least 100 spikes and to have a mean firing rate of at least 0.1 Hz for at least one of the six possible conditions (three tilt, 0 °, 15 °, 25 °; two slope directions, uphill and downhill). In addition, a place cell had to have a spatial information score (see below) of at least 1 bit/spike and spatial coherence (see below) > 0.5 for at least one condition. Data was pooled across animals, however, the general patterns described were consistent across all animals tested.

### 2.11 Place cell metrics

To determine the peak firing rate and place field size, the floor of the shuttle box was subdivided into 2.5 cm^2^ bins. An occupancy map based on the tracking data was then created based on the amount of time the rat spent in each bin. Bins with an occupancy time <100 ms were removed. A spike map was then created for each single unit based on the number of spikes which occurred in each bin. Elementwise division was used between the spike map and occupancy map to create a firing rate map where each bin contained the firing rate for a cell. The peak firing rate for a place cell was determined by the bin which had the highest firing rate.

A place field map was created for each cell based on the firing rate map. Place field maps utilized a firing rate criterion to remove bins where the cell was not substantially active in and/or did not display place field‐like activity. First, a Gaussian smoothing kernel was applied to the firing rate map with a 2.5 cm^2^ (1 sigma) smoothing window. Following this, each bin of the place field map was checked to see if it had a firing rate of at least 15% of the peak firing rate and had seven neighboring bins that also met this firing rate criterion. If a bin did not meet these criteria it was set to 0 on the place field map so it would not be included in the place field size calculation. Following this process of removing underactive bins, the number of distinct place fields was found using Matlab's bwlabel function for finding connected components. Afterward, each field was analyzed separately for its size (total bins with elevated firing), length, width, and aspect ratio (length/width). If a place cell had multiple fields, we chose the largest field to be its “main field.” All further place cell analysis described below was carried out on the unsmoothed firing rate maps (not the place field maps).

Spatial information measures the amount of information, in bits per spike, that a given spike conveys about the rat's location within an environment (Skaggs, Mcnaughton, Gothard, & Markus, 1993). The more spatial information a cell's spikes convey, the more that cell can be relied upon to decode the rat's position within the environment. The formula for spatial information is as follows:

$$Information=\overset{N}{\underset{i=1}{\sum }}p_{i}\frac{\lambda_{i}}{\lambda }log2\frac{\lambda_{i}}{\lambda }$$

where the environment is divided into *N* non‐overlapping bins with *i* = 1,…, *N, p*_*i*_ is the occupancy probability of bin *i*, λ_*i*_ is the mean firing rate for bin *i*, and λ is the overall mean firing rate of the neuron.

Sparsity was also measured for each place cell (Skaggs, McNaughton, Wilson, & Barnes, 1996). Sparsity is akin to information in that it measures the portion of the environment in which a cell is active. The formula for sparsity is:

$$Sparsity=\frac{\left[\lambda \right]}{\left[\lambda^{2}\right]}=\frac{{\left(\sum p_{i}\lambda_{i}\right)}^{2}}{\sum p_{i}\lambda_{i}^{2}}$$

where the square brackets [] denote the expected value average over all locations. All other symbols are as described for the Information equation.

Spatial coherence is a measure of how spatially concentrated a place cell's activity is (Muller & Kubie, 1989). Spatial coherence is measured by the average *z*‐transformed correlation of the firing rate of a given bin to the mean firing rate of the surrounding eight bins, carried out for every bin of the apparatus.

When analyzing the place cell metrics described above, only place cells which were active (met the place cell criteria) on a given condition contributed to that condition. If a place cell was not active on a given condition, its data was not included for that condition. For example, if a place cell was only active on 25 ° uphill, the metrics of its activity on 25 ° uphill were used, while the metrics for the other five conditions were not included in the calculation of those five condition's averages.

### 2.12 Place cell sequence plots

To visualize the activity of all the recorded place cells during a condition, sequence plots were created using firing rate maps. Sequence plots show the activity of many place cells by collapsing the short axis of the apparatus by averaging the firing rates of each short axis column along the long axis. Because of the narrow width of the shuttle box, there tended to be little deviation in place field width across the short axis. This transformation results in a one‐dimensional (1D) vector of 2.5 cm^2^ bins for the long axis of the apparatus. For our sequence plots, each row is a 1D vector of one place cell. Place cells can then be ordered based on the location of their place field, as determined by the bin with the peak firing rate. Place cell activity for any condition, say 15 °, may then be arranged based on their field location in the apparatus in the 0 ° condition. This method allows for the visualization of how much place cell activity changes when the apparatus is tilted from 0 ° to 15 °. For visual clarity the firing rates of each place cell were normalized to be between 0 (minimum firing rate) and 1 (max firing rate) for a consistent *z* axis. Firing rates for uphill plots and downhill plots were normalized separately.

### 2.13 Spatial activity correlations

While the place cell metrics described above capture how all active place cells were responding to tilt, we were also interested in how individual place cells were changing their activity due to changes in tilt. To test this, we utilized the occupancy maps for each place cell for each tilt condition and determined which bins were occupied on all three tilt conditions within a slope direction. Then, for each tilt condition the firing rate in each of the common‐occupied bins were turned into a 1D vector and correlated between each pair of tilt conditions. There had to be a minimum of three common‐occupied bins with non‐zero firing rates to avoid spurious correlation values. To determine the importance of spatial location, bin firing rates were randomly shuffled then correlated 10,000 times. The average correlation values from these 10,000 iterations were then compared to the actual correlation values.

### 2.14 Remapping

Remapping analyses were carried out by comparing how place cells changed their activity within or between tilt conditions while keeping slope direction (uphill/downhill), and thus running direction, constant. Only place cells that met the place cell criteria for one or both of the two conditions being compared were deemed “active” and underwent more granular remapping analysis. Remapping analysis was conducted on the change in activity from the shallower tilt angle to the steeper tilt angle. Place cells which were not active on the two conditions being compared were deemed “inactive.” Several different types of remapping were considered. Cells could “turn on” or “turn off” if they met the place cell criteria for one epoch but not the other (one type of complex remapping). For place cells which did meet the place cell criteria for both conditions in question, we determined if these cells field‐remapped (the second type of complex remapping), rate remapped, or remained stable. First we tested if a place cell field‐remapped by comparing the location of the bin with the maximum firing rate using the 1D place field maps of the two cells for both conditions. If their maximum firing rate bins differed by 20 cm, or ∼20% of the running area, the place cell “field‐remapped” in that its field location shifted from one epoch to the other. If the place cell did not field‐remap, it was then determined whether its firing activity differed significantly between the two conditions. The firing rates of each trial for one condition were tested against the trial firing rates of the other condition with a Wilcoxon rank sum test. If the firing rates significantly differed (*p* < 0.05) then the place cell was considered to have “rate remapped” between the two conditions. Finally, if the activity of the place cell did not meet any of these remapping criteria between a pair of conditions it was considered to be “stable.”

Further remapping analysis was carried out on place cells divided into “bottom” and “top” place cells depending on where their maximum firing rate was located in the maze in the 0 ° condition, with “top” being that half of the maze that was raised highest in the tilt conditions.

### 2.15 Phase precession analysis

To quantify how the timing of spikes relative to the underlying theta rhythm changed as animals moved through each place field, an analysis of phase precession (O'Keefe & Recce, 1993; Skaggs et al., 1996) was conducted. For phase estimation, the CA1 LFP was bandpass filtered between 7 and 9 Hz and the Hilbert transform was applied. The phase reference was always to the LFP in the CA1 pyramidal cell layer theta, and 0 ° corresponds to the trough in the negative portion of the filtered LFP. Place field position was determined automatically by dividing the shuttlebox into 4 × 20 pixels and selecting clusters of pixels that were in the region of the apparatus that excluded reward areas and where cells fired at above average firing rate and had at least two neighbors that also did so. Place fields were detected separately for each of the slope conditions and where more than one place field was found for a cell in a condition, data from the largest field was analyzed. All place field determination and data analysis were from data obtained as the animal ran in the same direction, either up the slope or on the flat.

For all spikes that occurred with a place field, spike phase was determined by matching animal position in the field to the instantaneous phase of the 7–9 Hz theta rhythm. The relationship between phase and position in each place field was measured using procedures described previously (Kempter, Leibold, Buzsaki, Diba, & Schmidt, 2012). Briefly, his involves using circular‐linear regression procedures to provide a robust estimate of the slope and phase offset of the regression line, and a correlation coefficient for circular–linear data that is a natural analogue of Pearson's product‐moment correlation coefficient for linear–linear data. This procedure gets around the potential problems associated with using linear‐linear correlation on circular data. The fits were constrained to have a slope of no more than ± 2 theta cycles per place field transverse. Previous studies indicate that phase precession occurs with a negative slope (O'Keefe & Recce, 1993). Phase precession analysis was conducted by combining spiking data from all passes through the place field for all cells that had a total of at least 50 spikes within the place field in the condition of interest and where the magnitude of the amplitude envelope of the filtered EEG, as derived from the Hilbert transform and tested for each spike at the time of firing, was above the mean. These constraints removed noise in the data potentially produced by low firing‐rate cells or spikes that occurred when EEG amplitude was low and therefore phase determination might be problematic. Analysis of firing, phase‐position slope and correlation data was conducted using a between subjects ANOVA on individual cell data. The phase offset data, which corresponded to the theta phase at which spiking occurred as the animal entered the field was compared across conditions using circular statistics, including the Watson‐Williams test for comparison of circular data (Zar, 1999).

### 2.16 Histology

After completion of behavioral testing the placement of the tetrodes were confirmed by creating a lesion at the tip of each tetrode by passing 2 mA of current for one second on two wires of each tetrode while the rat was deeply anesthetized with isoflurane. Rats were subsequently overdosed on isoflurane in a large bell jar and perfused transcardially with 120 mL of 0.9% saline followed by 120 mL of 10% formalin in saline. Brains were removed and placed in 30% sucrose solution until they sunk. Brains were then frozen and sliced with a microtome (Lecia Biosystems, LLC) to 60‐µm‐thick coronal sections. Sections were mounted and stained with thionine acetate (Santa Cruz Biotechnology) and tetrode placement was confirmed with a lower power (1.5×) digital microscope (Lecia Biosystems, LLC) and tetrode movement logs.

## 3 RESULTS

### 3.1 Rodent behavior and place cell properties

We recorded single units from the dorsal CA1 subregion of well‐trained rats as they shuttled back and forth in a shuttle box which could be laid flat (0 °) or tilted to 15 ° or 25 ° to manipulate tilt (Figure 1a). Behavioral and neurophysiological data were only analyzed in the running region of interest with the rewarded endzone regions excluded (Figure 1b). Data was collected over six consecutive days, with counterbalancing for tilt condition presentation order and which side was elevated (Figure 1c). The rats completed an average of 18.8 (Standard Error of Mean; SEM ± 0.17) successful trials (i.e., did not turn around, took <7 s) for each tilt angle‐slope direction (tilt angles: 0 °, 15 °, and 25 °; slope directions: uphill and downhill). Rat's running speeds were significantly influenced by tilt (*F* (2, 74) = 19.88, *p* < 0.0001), slope direction (*F* (1, 37) = 9.897, *p* = 0.0033), and their interaction (*F* (2, 74) = 10.33, *p* < 0.0001; Figure 1d). On average, rats slowed down with an increasing tilt angle, running an average speed of 33.1 cm s^−1^ (SEM ± 0.9) on 0 °, 32.1 ± 0.8 cm s^−1^ on 15 °, and 28.3 ± 0.7 cm s^−1^ for 25 °. Surprisingly, rats were significantly faster on 15 ° (33.4 ± 1.1 cm s^−1^) and 25 ° (30.0 ± 1.0 cm s^−1^) uphill than on 15 ° (31.5 ± 1.2 cm s^−1^; (Tukey's; *q* (74) = 5.363, *p* = 0.004) and 25 ° (26.6 ± 1.0 cm s^−1^; Tukey's; *q* (74) = 6.98, *p* < 0.001) downhill, respectively. Running speeds on 0 ° “uphill” (32.7 ± 1.2 cm s^−1^) and “downhill” (33.4 ± 1.2 cm s^−1^) did not differ significantly (Tukey's; *q* (74) = 1.58, *p* = 0.874). Anecdotally, rats tended to employ a fast hopping‐like gait when travelling uphill and a more cautious walk for downhill runs. Tilt also significantly affected (*F* (2, 74) = 13.51, *p* < 0.001) the way in which rats travelled in the shuttle box; as the tilt angle increased, rats ran more irregular routes as observed by the total number of 2.5 cm^2^ bins they occupied in a given condition (slope direction: *F* (1, 37) = 0.8214, *p* = 0.3706); interaction: *F* (2, 74) = 2.427, *p* = 0.0953; Figure 1e). Rats utilized significantly more area to run on 25 ° compared to 0 ° (Tukey's; *p* < 0.0001) and 15 ° (*p* = 0.0001). In conjunction with previous physiological studies on rats running inclined and declined treadmills (Armstrong et al., 1983; Brooks & White, 1978; Chavanelle et al., 2014), the reduction in running speed and more irregular paths likely indicate the difficulty of travelling on sloping terrain.

### 3.2 The effects of tilt on place cell encoding

A total of 225 putative single units were recorded across all recording sessions from the seven rats. Of those cells, 99 met our strict place cell criteria for inclusion in subsequent analyses with an average of 14.1 ± 4.0 place cells (PCs) recorded per rat (Rat 1, 25; Rat 2, 4; Rat 3, 1; Rat 4, 6; Rat 5, 18; Rat 6, 17; Rat 7, 28). We were interested in whether there were systematic changes to standard measures of place cell activity as our apparatus was tilted. Overall, tilt and slope direction appeared to have little effect on most measures of place cell activity (Table 1). Two‐way ANOVAs with tilt (0 °, 15 °, 25 °) and slope direction (uphill, downhill) as factors revealed no significant difference (*p* > 0.05) for either factor or their interaction when comparing mean firing rates, peak firing rates, spatial information, or spatial coherence. Tilt angle did have a small but significant effect (*F* (2, 206) = 3.056, *p* = 0.0492) on the sparsity of place cell firing (slope; (*F* (1, 206) = 0.414, *p* = 0.5204) interaction; (*F* (2, 206) = 0.565, *p* = 0.5692)). Tukey's test for multiple comparisons revealed a significant difference between the average sparsity of place cell activity on 0 ° compared to 25 ° (Tukey (206) = 3.401, *p* = 0.0448), and no differences between 0 ° to 15 ° and 15 ° to 25 ° (*p* > 0.05). Because of the differences in the number of bins rats occupied across conditions, we measured place field sizes as a percentage of occupied area covered by the place field (place field size/total bins occupied). Neither tilt nor slope direction affected the number of fields place cells had, the total coverage of all place fields, or the size or aspect ratio of a cell's main place field (*p* > 0.05). Furthermore, infield firing rates and outfield firing rates did not differ across tilt or slope conditions (*p* > 0.05).

**Table 1:** Place cell metrics across tilt‐slope direction conditions

	Uphill			Downhill		
	0 °	15 °	25 °	0 °	15 °	25 °
Firing rate (Hz)	3.31 ± 0.25	3.63 ± 0.37	3.58 ± 0.38	3.81 ± 0.26	3.64 ± 0.33	3.23 ± 0.25
Peak firing rate (Hz)	32.7 ± 2.4	33.5 ± 3.4	36.6 ± 3.2	33.7 ± 2.3	36.5 ± 2.7	35.4 ± 2.5
Information score (bits/spk)	1.62 ± 0.08	1.57 ± 0.07	1.68 ± 0.06	1.62 ± 0.07	1.72 ± 0.08	1.71 ± 0.07
Sparsity^a^	0.24 ± 0.01	0.25 ± 0.01	0.22 ± 0.01	0.25 ± 0.01	0.23 ± 0.01	0.22 ± 0.01
Spatial coherence (*r*)	0.90 ± 0.04	0.98 ± 0.05	0.94 ± 0.05	1.10 ± 0.06	1.01 ± 0.05	0.94 ± 0.04
Number of place fields	1.30 ± 0.10	1.33 ± 0.12	1.23 ± 0.08	1.21 ± 0.07	1.21 ± 0.06	1.25 ± 0.07
Total fields/occupancy (%)	27% ± 2%	24% ± 2%	26% ± 1%	24% ± 1%	24% ± 1%	23% ± 2%
Main field/occupancy (%)	26% ± 2%	22% ± 1%	25% ± 2%	23% ± 2%	23% ± 1%	22% ± 1%
Place field aspect ratio	3.23 ± 0.32	3.13 ± 0.33	3.09 ± 0.40	3.31 ± 0.23	2.89 ± 0.16	2.85 ± 0.17
Infield firing rate (Hz)	12.4 ± 1.26	14.2 ± 2.01	14.1 ± 1.33	15.0 ± 1.31	15.8 ± 1.53	14.2 ± 1.34
Outfield firing rate (Hz)	1.08 ± 0.11	1.32 ± 0.14	1.18 ± 0.15	1.18 ± 0.09	1.16 ± 0.13	1.13 ± 0.10

a indicates significant effect (*p* < 0.05) for tilt or slope direction (see text for details).

### 3.3 Place cells remap in response to tilt

**Figure 2:**
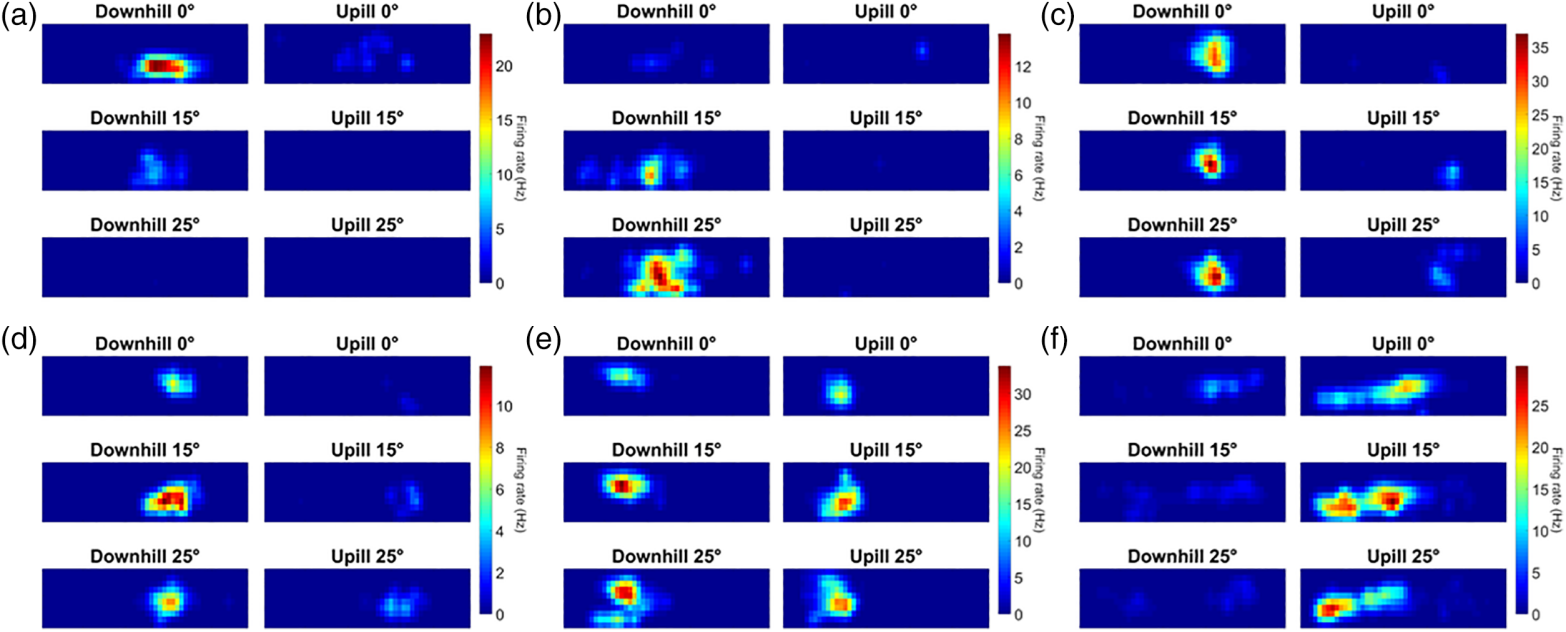
Six example place cell firing rate maps (a–f), with firing of each cell illustrated across all six conditions. Every subplot shows the experimental apparatus as a series of 2.5 cm^2^ bins with the *x* and *y* axes corresponding to position in the shuttle box. The *z*‐axis is the firing rate of the cell in spikes per second (Hz) for each bin where warmer colors indicate a higher firing rate. For each place cell, the firing rate color scale across the three conditions is determined by the highest peak firing rate of the six tilt conditions. The left column of a plot shows Downhill runs while the right column shows Uphill runs. Each row of a plot corresponds to one of the three tilt conditions; top, 0 °; middle, 15 °; bottom, 25 °. Note that several cells fired specifically for one or two tilt conditions (e.g., a and b) [Color figure can be viewed in the online issue, which is available at http://wileyonlinelibrary.com.]

**Figure 3:**
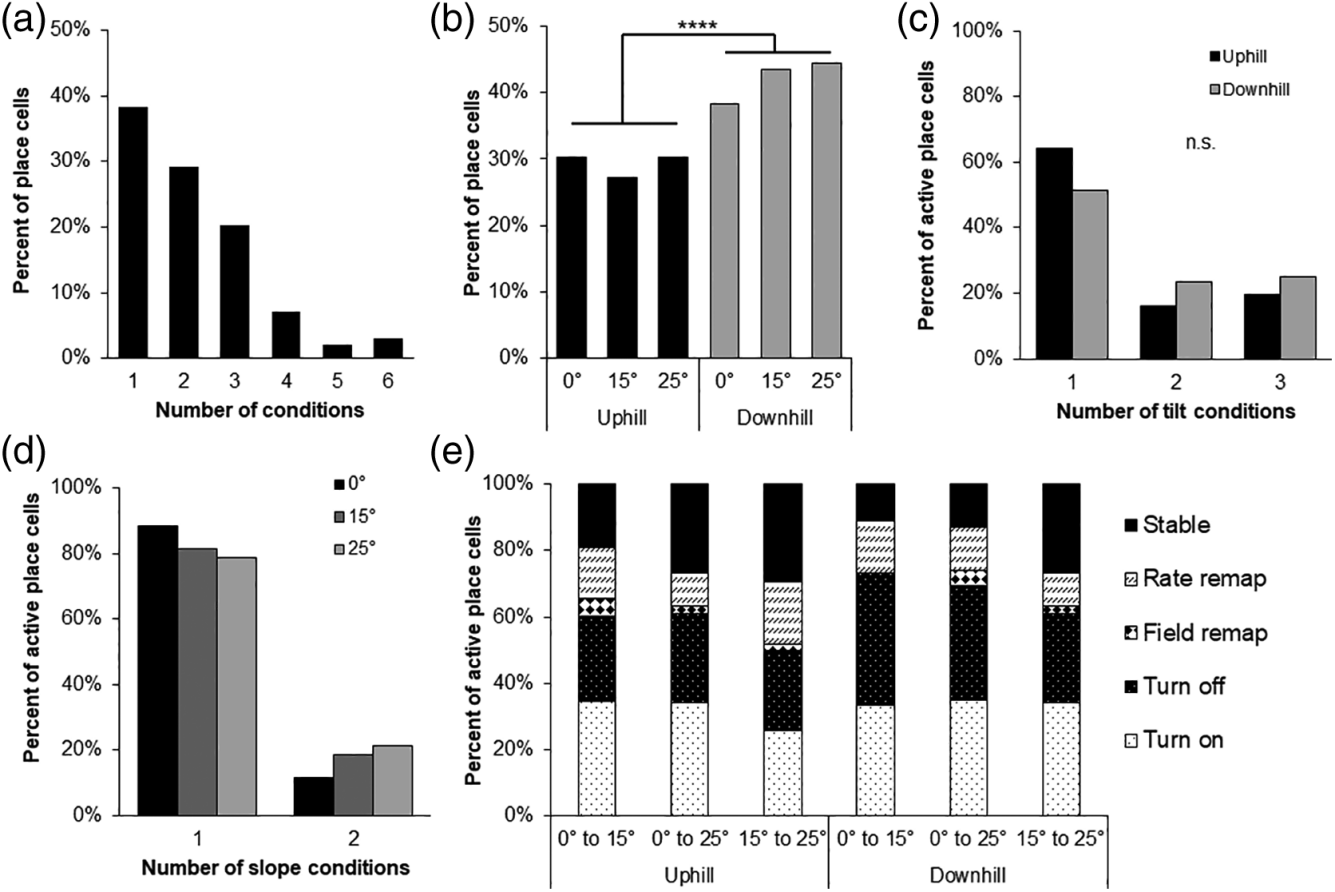
Place cell encoding of tilt conditions. (a) The number of tilt‐slope direction conditions place cells were active for. (b) The percent of place cells active for each tilt‐slope direction condition. (c,d) The number of tilt conditions (c) or slope direction conditions (d) place cells were active for. (e) Types of remapping observed between pairs of tilt conditions within a slope direction comparing shallower angles to steeper angles

Previous studies have demonstrated that place cells will alter their activity, or “remap” in response to manipulations to an environment, such as changes to the shape of environments or visual cue locations (Muller & Kubie, 1987). We were initially interested in how place cell activity was remapping in response to changes in tilt. A diverse range of remapping responses to the tilt manipulation were observed from place cells recorded in different animals (Figure 2). Most place cells met the place cell criteria for either one (38% Figure 3a; e.g., Figure 2a), two (29%; e.g., Figure 2b), or three of the slope x direction conditions (20%; e.g., Figure 2c,d,f), with very few meeting the criteria for four (7%), five (2%), or all six (3%; e.g., Figure 2e). On average, cells met the place cell criteria for 2.1 conditions (SEM ± 0.12). There was no significant difference in the number of place cells active for a given tilt angle (0 ° = 78, 15 ° = 70, 25 ° = 74; *X* ^2^ (2) = 0.27, P = 0.867), however there were significantly more place cells active on downhill conditions (n = 125) versus uphill conditions (*n* = 87; *X* ^*2*^ (1) = 6.81, *p* = 0.009; Figure 3b). During both uphill and downhill conditions, most place cells were active on one of the three tilt conditions, with fewer active on two or three condition (Figure 3c). The number of tilt conditions place cells were active for did not differ significantly between uphill and downhill conditions (*X* ^2^ (2) = 2.12, *p* = 0.333). Most place cells (71%) exhibited directional selectivity (Figure 2a–d,f) and only met the place cell criteria for one slope direction (Figure 3d). In contrast, 29% of place cells showed bidirectional activity and were active on both uphill and downhill runs (Figure 2e). There were no significant differences in the number of unidirectional versus bidirectional place cells across the tilt conditions (*X* ^2^ (2) = 2.22, *p* = 0.329). Taken together, place cells tend to be unidirectional and selectively active on specific tilt‐slope direction conditions.

### 3.4 Place cell remapping across conditions

We wanted to further quantify the types of remapping cells were undergoing as the tilt of the environment was manipulated. We analyzed whether or not place cells remapped between conditions and if they did remap, what type of remapping they underwent. For each place cell, we asked how it was changing its activity between each pair of tilt conditions while keeping slope direction constant (Figure 3e). A Chi‐squared test determined there were no differences in the number of place cells undergoing rate or field remapping, turning on or off, or remaining stable across the condition pairs (*X* ^2^ (25) = 29.29, *p* = 0.252). Because there were no differences, we will present the average percentage of place cells across the six condition pairs which underwent each type of remapping. On average, 51% ± 3% of recorded place cells were inactive between a given pair of tilt conditions. If a place cell was active on two conditions, complex remapping was the most common form of activity change with place cells either turning on (16% ± 1%) or shutting off between conditions (14% ± 1%). Field remapping was quite rare, with an average of just 1.3% ± 0.4% cells remaining active on two conditions but with distinct place field locations. Rate remapping, where place cells have a stable field location but significantly alter their firing rate between two conditions, was more common with an average of 7% ± 1% place cells. Lastly, an average of 10% ± 2% of place cells had stable activity between two conditions. These results further indicate that the degree of change between tilt angles does not have an effect on the magnitude or type of place cell remapping. Rather, any change to the tilt of an environment results in place cell ensembles undergoing consistent but substantial partial‐complex remapping.

### 3.5 Place cell sequence plots

To visualize the place cell remapping that was occurring across tilt conditions we generated a series of sequence plots. Here, all 99 place cells that were active for at least one slope direction‐tilt condition (tilt: 0 °, 15 °, and 25 °; slope direction: uphill and downhill) were included in the plots. For each place cell, the firing rates across the apparatus in all conditions is displayed as heat maps and for each condition. Place cells are ordered according to their place field position in the apparatus using one tilt × slope direction condition as a baseline and plotting the other conditions relative to this baseline.

**Figure 4:**
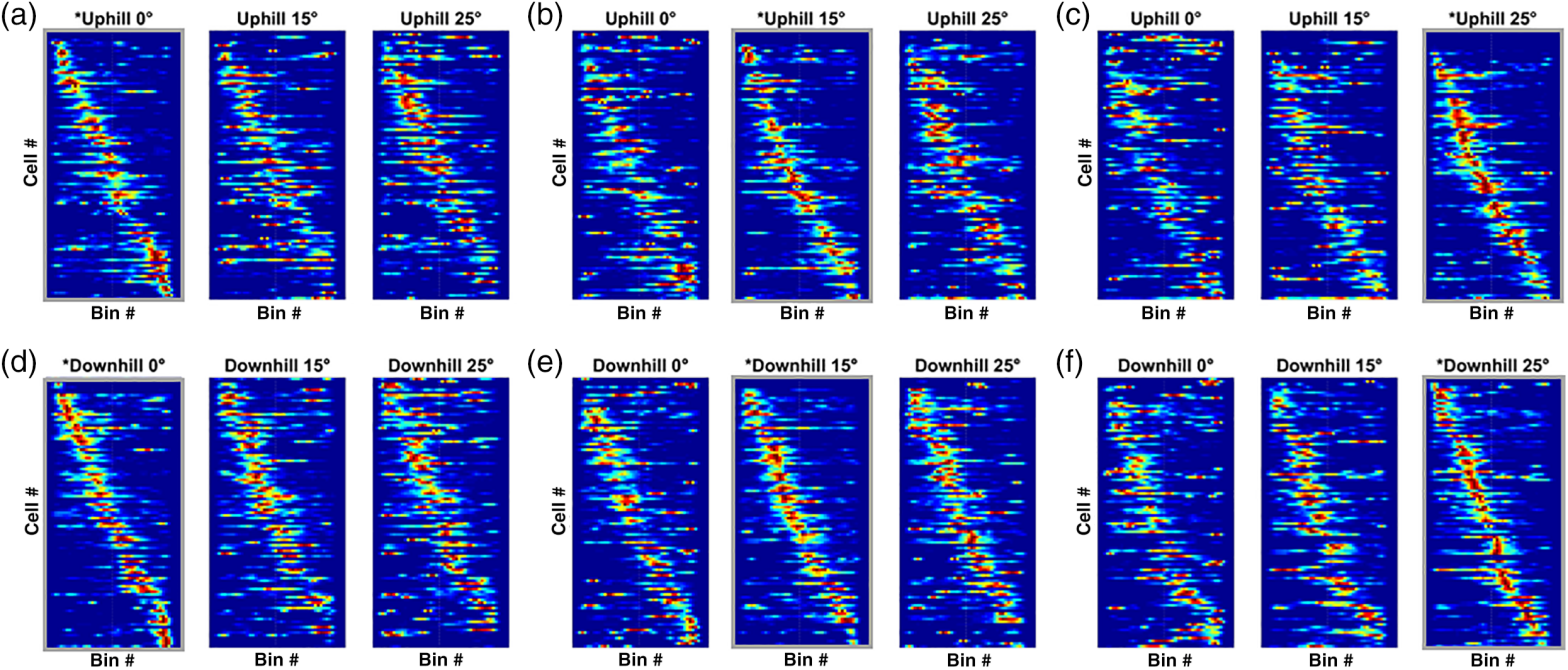
Place cell sequence plots. Thus, the *x* axis represents the longitudinal extent of the apparatus and each row of the *y* axis is a place cell. Place cells are ordered based on their field location of one of the three tilt conditions (0 °, 15 °, and 25 °; from left to right) which served as the baseline. The baseline condition is indicated by a grey border and an asterisk in the title. The *z* axis is a cell's normalized firing rate; warmer colors represent a higher firing rate. Changes to the tilt have a substantial complex remapping effect on place cells as evidenced by the number of cells that turn on or off with a change in condition. Cells that are active for multiple tilt conditions generally have a stable place field location in the maze as indicated by the preserved place field location sequence across changes in tilt [Color figure can be viewed in the online issue, which is available at http://wileyonlinelibrary.com.]

Sequence plots were created and ordered according to the place field sequence order for all three tilt conditions (0 °, 15 °, 25 °), separately for each slope direction (uphill or downhill) (Figure 4). A grey outline of the sequence plot and asterisk in the condition title indicates which condition is being used to organize the place cells by their field location in that condition. Changes in the tilt of the apparatus results in a wide range of remapping activity with some place cells turning on or off while others remain active across tilt conditions with changes to their field location or firing rate. Overall, place field sequences tend to hold their ordered sequence across the different tilts, suggesting that at least some place cell fields are stable on different tilt angles. We also ordered place cells from one slope direction, and thus running direction, to the other which showed a near total breakdown in place field sequence across the environment (data not shown). Thus, tilting a fixed environment causes substantial, partial‐complex remapping of place cell populations for both uphill and downhill trajectories.

### 3.6 Place cell activity across tilt and slope conditions

**Figure 5:**
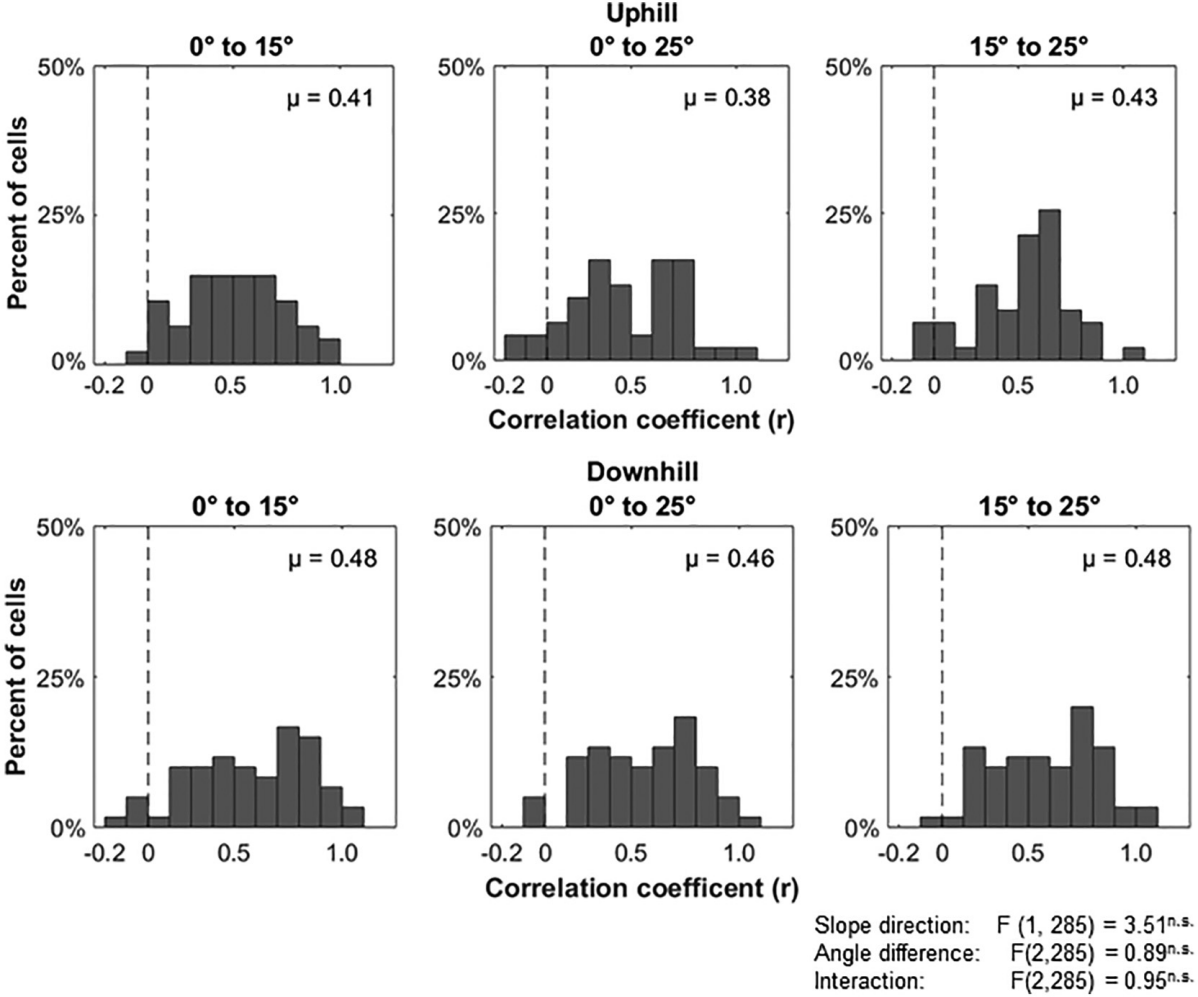
Place cell activity correlations across tilt‐slope direction conditions. All plots show a histogram of correlations values of place cell activity between pairs of conditions. The dotted line is the average correlation value from shuffling the location of firing rate bins. No significant differences were found in the difference in tilt angle to the degree of place cell activity difference on uphill or downhill runs

To quantify the effects of tilt shown in the sequence plots, a spatial correlation analysis was utilized to test how individual place cells were being affected by changes in tilt and slope direction. We hypothesized that place cells may use tilt angle as a way to discriminate between experiences. If this is so, conditions where the tilt angle is more similar (15 ° to 25 °; 10 ° difference) should be more correlated to each other compared to conditions where the tilt angle is more different (0 ° to 25 °; 25 ° difference). For each place cell we computed a correlation comparing the firing rates of each bin commonly‐occupied between the two conditions, for every tilt‐slope direction pair. We then aggregated the correlation values from every place cell for each pair of conditions (Figure 5). A two‐way ANOVA showed no significant effect for slope direction (*F* (1, 285) = 3.51, *p* = 0.0620), the difference in tilt angle between tilt pairs (*F* (2, 285) = 0.8864, *p* = 0.4133), or their interaction (*F* (2, 285) = 0.0500, *p* = 0.9516). We further tested these data against correlation values generated from randomly shuffling firing rate bin locations for each condition pair. For all six pairs of conditions, the actual correlation values were significantly greater from those that would be generated by chance if spatial specificity was irrelevant (Wilcoxon rank sum test; *p* < 0.001). These data suggest that overall dCA1 place cells treat each tilt condition as a unique environment and form distinct maps for each condition, however, these maps are not completely unrelated.

### 3.7 Place cell remapping between and within conditions

To confirm that the place cell activity changes apparent across the different tilt conditions were due to changes in tilt and not simply due to changes in place cell activity over time, we compared within‐tilt condition changes with those observed between different tilt conditions across similar time windows. Place cell activity was compared within the second and third tilt conditions for each recording session by analyzing how place cells remapped from the first 10 trials to the second 10 trials. To test the effect of changing tilt, the latter 10 trials from the first tilt condition were compared to the first 10 of the second condition and the latter 10 of the second to the first 10 of the third. When place fields were characterized by the type of remapping observed across these two comparisons, it was apparent that remapping was quite different in the tilt‐change comparison compared to the within‐condition situation (*X* ^2^ (4) = 23.5, *p* < 0.001, Chi‐squared test; Table 3). We further combined all forms of remapping into one category to test against stable place cells. Place cells were significantly more likely to remain stable within a tilt condition compared to between tilt conditions (*X* ^2^ (1) = 16.6, *p* < 0.001). Together, these data indicate that the change in place cell response across tilt angles cannot simply be explained as instability over time.

### 3.8 Elevation change has no effect on remapping

We next set out to test whether or not the tilt‐associated remapping we were observing was related to the tilt per se or to the vertical transition in space that occurred across most of the apparatus as it was shifted from the flat to tilted condition. We hypothesized that if place cells were encoding the elevation of the apparatus it would be expected that the further a place field moved through 3D space (primarily vertically) the more likely it would be for a place cell to remap. To these ends we divided the apparatus up into two halves with the knowledge that overall one half (high) was shifted through space to a greater extent than the other half (low) when the apparatus was shifted from the flat to the sloped condition (see Figure 1a). We first considered all place cells which met the place cell criteria for the 0 ° tilt condition in either slope direction. The place cell's field location in the bottom or top of the apparatus was determined from the bin of the 1D place field map that had the maximal firing rate. For those place cells that were active in the 0 ° condition we then asked how changes in elevation altered their activity. We discovered that place cells with fields on the low half of the apparatus were just as likely to remap (turn on/off/field/rate) or remain stable as place cells with fields on the high half of the apparatus (*X* ^2^ (1) = 1.635, *p* = 0.201; Table 2). Differentiating between complex remapping (turn on/off, field remap), rate remapping, and stable place cells also shows no significant effect for a field's vertical transition (*X* ^2^ (2) = 2.89, *p* = 0.236). This analysis was also repeated using a place cell's center of mass location rather than maximal firing rate location to determine the top/bottom categorizing factor, with no difference in results (*p* > 0.05). Thus, remapping is likely driven directly by the change in the slope, with cells remapping to encode a particular whole‐tilt “context,” rather than being an effect of the vertical transition of part of the apparatus.

**Table 2:** Frequency of remapping types observed in place cells between 0 ° and tilt conditions (15 ° and 25 °) based on main place field location within the apparatus

	Top	Bottom
Inactive	44	45
Turn on	17	24
Turn off	24	11
Field remap	3	2
Rate remap	4	7
Stable	6	11

**Table 3:** Frequency of remapping types observed in place cells between 0 ° and tilt conditions (15 ° and 25 °) based on main place field location within the apparatus

	Within	Between
Inactive	379	228
Turn on	36	60
Turn off	55	45
Field remap	4	4
Rate remap	13	10
Stable	107	49

### 3.9 Phase precession analysis

**Figure 6:**
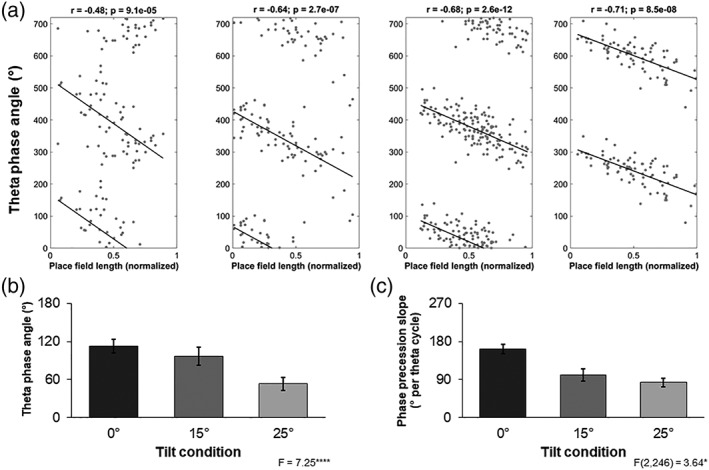
Phase precession. (a) Examples of the phase precession of place cell spiking against theta activity as the animal traverses the place field from left to right. Zero degrees 0 ° corresponds to the trough in the negative portion of the filtered LFP recorded at the CA1 cell layer. Data points are reproduced twice over two theta cycles. The left plot is recorded from an animal moving on a flat surface while the middle two are tilted at 15 ° and the rightmost, 25 °. (b) Place cell firing phase, with reference to the underlying local theta as the animal enters the place field, is systematically shifting to earlier in the cycle as the apparatus is tilted. Data are mean angle and circular sem. (c) Phase precession slope decreases as the apparatus is tilted. The data are degrees per cycle

Data for phase precession analysis was gathered from 78 cells that met the sampling criteria at zero degrees of tilt, 79 cells at 15 degrees and 92 cells at 25 degrees (Figure 6a). All analysis was conducted for travel up the tilted surface or for the equivalent direction on the flat. A quantification of phase precession characteristics was provided through the circular‐linear correlation procedure, which indicated no difference between tilt conditions in terms of the proportion of cells that generated statistically significant (*p* < 0.05) circular‐linear fits (*X* ^2^ = 4.91, *p* = 0.09). Overall, there was also no difference in the circular linear correlation coefficient calculated for the three different slope conditions (*F* (2,245) = 1.359, *p* = 0.259. However, a comparison of the best fit line through the data indicated some differences between tilt conditions. For all groups, the initial firing as the animal entered the place field tended to occur on the rising phase of the theta cycle as we recorded it at the CA1 pyramidal cell layer, equivalent to firing occurring just after the peak of theta at the fissure as described previously (Skaggs et al., 1996). There was, however, a significant shift towards an earlier firing phase as the tilt of the apparatus increased (Rayleigh's *F* = 7.25, *p* < 0.001; Figure 6b). A comparison of the slope of the best‐fit to the data measuring the precession across the theta cycle indicated that slope decreased to become less‐negative as the tilt of the apparatus increased (*F* (2,246) = 3.64, *p* = 0.028; Figure 6c). These changes in phase precession did not appear to be artifacts of other changes in cell firing or animal movement as no significant between‐tilt differences were observed in mean firing rate within the field, place field width, place field position, theta frequency, theta amplitude or animal speed within the field (all *p* > 0.1).

## 4 DISCUSSION

To understand how a change in the slope of a traversed surface influenced the hippocampal representation of space, we analyzed the activity of dCA1 place cells as rats shuttled back and forth in a high‐sided box tilted at 0 °, 15 °, and 25 ° angles. Our data indicated that place cells had no loss of spatial specificity on tilted environments as demonstrated by standard place cell metrics. Nevertheless, place cells are sensitive to changes to the tilt angle of an environment. We showed that any change to the tilt of the shuttle box led to substantial partial remapping of place fields. However, the magnitude of the difference in tilt angle between conditions did not reflect the degree of remapping observed. In addition, the probability of a place cell remapping was not affected by how far the animal moved in the vertical dimension of space which supports the proposal that rodent place cells encode space anisotropically (Jeffery et al., 2013).

A well‐studied characteristic of hippocampal place cells is their ability to “remap” their activity in response to changes to the environment (Colgin, Moser, & Moser, 2008; Leutgeb et al., 2005; Muller & Kubie, 1987). Most commonly, changes to the shape or size of the environment or the locations of prominent cues can result in alterations in place cell firing, including changes in firing rate or changes in the location of the place field. Place cell remapping to changes in the tilt of an environment have, however, seldom been investigated despite previous studies showing that place cells respond to changes in vestibular information (Russell et al., 2003; Stackman et al., 2002) and can use slope as an orienting cue (Jeffery et al., 2006). Our data showed that a high proportion of place cells remapped as the tilt of the shuttle box was manipulated. As a result, many cells were only responsive to one or two of the tilt slope‐direction conditions; demonstrating the sensitivity of place cells to slope terrain. However, place cell encoding does not seem to be coupled to changes in slope angles as irrespective of whether the shuttle box slope was altered by 10 ° (15 ° to 25 °) or 25 ° (0 ° to 25 °), similar levels and types of remapping were observed. These data indicate that the hippocampus is encoding each tilt condition as a discrete context with terrain slope as a differentiating cue.

A subset of place cells did remain active, with a stable place field location and firing rate, on more than one tilt condition. These place cells may aid in associating together these experiences (Eichenbaum, 2004; Leutgeb, Leutgeb, Treves, Moser, & Moser, 2004; McKenzie et al., 2014). Stable place cells, especially those active on two tilt conditions within a slope direction, may accomplish this associative function by having a broader terrain slope tuning curve than other cells. An alternative, and not mutually exclusive, explanation is that subsets of place cells are utilizing different reference frames for their spatial specificity (Gothard et al., 1996; Knierim & Hamilton, 2011; Wiener, Korshunov, Garcia, & Berthoz, 1995; Zinyuk, Kubik, Kaminsky, Fenton, & Bures, 2000). These stable place cells, especially those active on all three tilt conditions within a slope direction, may be driven by egocentric, path integration information which is resilient to changes in terrain slope and remain stable when terrain slope is altered.

To our knowledge only one previous study has investigated how place cells respond to tilting an environment (Knierim & McNaughton, 2001). Knierim and McNaughton (2001) showed that when part of a square track was tilted from 0 ° to 45 °, partial remapping occurred. No consistent change in other metrics, such as peak firing rate, was found. In this previous study, however, the track had no side walls and was located such that animals had a clear view of distal visual cues in the recording room. For this reason, we cannot be sure if the remapping observed in this previous study was a result of the tilt itself, or a response to the apparent shift of the distal cue locations that would have accompanied the track manipulation. In contrast, in the present study we have endeavored to minimize the influence of the tilt manipulation on distal cues by depriving the animal of any visual clues that might have become associated with a tilt condition. Our remapping findings are therefore consistent with those previously reported by Knierim and McNaughton (2001), but further constrain interpretations of the effect tilt has on place cell encoding.

A recent study by Hayman, Casali, Wilson, & Jeffery (2015) found that medial entorhinal (MEC) grid cell activity was disrupted between flat and tilted (40 °) terrain. Primarily, grid cells had decreased spatial coherence and lower symmetry with larger and more numerous fields. We had hypothesized that hippocampal place cell activity may also be disrupted on tilted terrain in a similar fashion because experimental and computational evidence has demonstrated that the MEC and grid cells are an important source of information for hippocampal place cells (McNaughton, Battaglia, Jensen, Moser, & Moser, 2006; Ormond & McNaughton, 2015; Savelli & Knierim, 2010). We did not observe any significant changes in place cell activity across our three tilt conditions. It may be possible that place cells activity could be disrupted on very steep slopes, such as the one used by Hayman et al. (2015), and that 25 ° is not sufficient to disrupt encoding. However, our findings are in line with recent experimental (Miao et al., 2015; Rueckemann et al., 2016) and computational models (Azizi, Schieferstein, & Cheng, 2014) which show that hippocampal place cell activity can be resilient to disruptions to the MEC. Hippocampal units may rather rely on other, non‐MEC spatial inputs, such as head direction (Stackman & Taube, 1998; Taube, Muller, & Ranck, 1990) and border cells (Lever, Burton, Jeewajee, O'Keefe, & Burgess, 2009), to generate the spatial selectivity of place cells (Bush, Barry, & Burgess, 2014).

Because our tilt procedure involved pivoting the shuttle box around one of its ends (Figure 1), the shift in the vertical position of any location in the apparatus during a tilt manipulation depended on the distance from that location to the pivot point. We used this difference to allow for an investigation of the remapping of place fields in the half of the box close to the pivot (small vertical shift) compared to those in the half distal to the pivot (large vertical shift). If the hippocampal representation of space in rats were volumetric and isotropic, as suggested by the discovery of 3D, spherical place fields in freely flying bats (Yartsev & Ulanovsky, 2013), one might anticipate that place cells with fields in the half of the box that had the greatest vertical movement through space would have a higher likelihood of remapping as the animals were shifted out of, or into, the vertical confines of particular place cell fields. Our analysis indicated, however, that the propensity for a place cell to remap was not affected by the half in which the cell's field was located, suggesting that in surface‐travelling‐mammals, such as rats, representations of space both by hippocampal place cells (Hayman et al., 2011) and entorhinal grid cells (Hayman, Casali, Wilson, & Jeffery, 2015) are planar and anisotropic (Jeffery et al., 2013).

An analysis of phase precession processes indicated that there were systematic changes to the way that spike firing related to the underlying local theta rhythm as tilt changed. In particular, firing began earlier in the theta cycle when the animal was entering a place field on a tilted surface. Furthermore, the amount of phase precession decreased as the slope increased. These effects could not be explained as artifacts of changes in variables such as theta frequency or amplitude, or animal running speed. It is unclear what function, if any, this change represents, however, it is possible that it might alter how the hippocampus “interpreted” the environment. Previous studies suggest that phase precession provides a constant “look‐ahead” function that allows for the planning of future trajectories (Skaggs et al., 1996; Wikenheiser and Redish, 2015). At any particular moment, a decrease in the slope of phase precession would compress the components of these trajectory predictions into a narrower time window. A potential consequence of this change is the enhancement of plasticity between cells representing distal regions through the opening of a temporal window for synapse potentiation that might not exist when spikes are temporally more distant (Dan & Poo, 2004). One result of this effect might be an expansion of place field size as cells gain greater influence over the firing of their distal (in terms of place field) neighbors. We did not see evidence of this expansion, although it has previously been observed to co‐occur with reduced phase precession slope (Shen, Barnes, Mcnaughton, Skaggs, & Weaver, 1997; Terrazas et al., 2005). It is possible, however, that such an expansion effect might only occur during the initial exposure to an environment, and then influence subsequent responses to other similar environments, such that in our well‐trained animals, the expression of this effect occurred on all slopes. This could be tested in future studies by only exposing animals to one slope condition and then examining the consequences on place field size. If an initial exposure to a novel slope does produce an expansion of place field size then this might lead to a perception that sloped surfaces extend further than they actually do (Proffitt, Stefanucci, Banton, & Epstein, 2003; Stefanucci, Proffitt, Banton, & Epstein, 2005; Witt, Proffitt, & Epstein, 2004). It is tempting to speculate that this might underlie a neural instantiation of Naismith's rule that slopes will take longer to traverse relative to the same distance on flat ground, although further studies will be required to determine whether this is so.

Overall, we have observed that a subset of hippocampal place cells are sensitive to changes to terrain slope. The encoding of terrain slope is a vital element of efficient navigation allowing an organism to avoid the time and energy costs associated with traveling uphill (Armstrong et al., 1983; Brooks & White, 1978; Chavanelle et al., 2014; Hoogkamer et al., 2014; Margaria et al., 1963; Minetti et al., 2002). Additionally, our findings contribute to the wider field of cost‐benefit analysis in the context of spatial navigation. Growing evidence has shown that place cells respond to the value of an experience (Allen, Rawlins, Bannerman, & Csicsvari, 2012; Ambrose, Pfeiffer, Correspondence, & Foster, 2016; Cheyne, 2014; Gauthier & Tank, 2017; McKenzie et al., 2014). Our data show that there are more place cells active on downhill runs versus uphill runs on the tilt conditions. This is unlikely to be due to speed differences, which if anything, would produce the opposite effect, with the slower downhill movement usually associated with reduced firing. Rather, this may indicate that the downhill route may have a greater relative value (benefit minus effort cost) and so has a larger ensemble of place cells representing it. Indeed, previous studies have shown that place cells over‐represent goal locations (Cheyne, 2014; Hollup et al., 2001) and preferred routes (Mamad et al., 2017).

During decision making, possible behaviors as well as their remembered values may be sent to downstream structures through the reactivation of place cell ensembles via sharp‐wave ripple replay events (Jadhav, Kemere, German, & Frank, 2012; Pfeiffer & Foster, 2013; Singer, Carr, Karlsson, & Frank, 2013) or theta sequences (Johnson & Redish, 2007; Wikenheiser & Redish, 2015). Our findings that place cells can encode terrain slope may aid in providing downstream cortical structures, such as the anterior cingulate cortex (Remondes & Wilson, 2013, 2015), not only with previous and possible routes through an environment but with effort information associated with those routes (Cowen, Davis, & Nitz, 2012; Hillman & Bilkey, 2010, 2012). As a result, prefrontal regions may selectively retrieve and reactivate the highest value hippocampal representations (Ito, Zhang, Witter, Moser, & Moser, 2015; Navawongse & Eichenbaum, 2013; Preston & Eichenbaum, 2013), resulting in the further differentiation of hippocampal ensembles based on value.
